# Influence of the Number of Inserts Used for Face Milling on Cutting Forces and Surface Roughness

**DOI:** 10.3390/ma17246052

**Published:** 2024-12-11

**Authors:** Cyril Horava, Martin Reznicek, Martin Ovsik

**Affiliations:** Faculty of Technology, Tomas Bata University in Zlin, Vavreckova 5669, 760 01 Zlín, Czech Republic; c_horava@utb.cz (C.H.); mreznicek@utb.cz (M.R.)

**Keywords:** end milling, flat surface, inserts, cutting forces, roughness

## Abstract

This article examines the effect of the number of inserts in a milling head on cutting forces during machining and the resulting surface roughness. An experimental study was used to compare results using different insert configurations while maintaining a constant feed per tooth. The resulting cutting forces and surface roughness were analyzed and discussed in the context of the optimal setting of cutting conditions. It was found that a reduced number of inserts does not necessarily lead to a reduction in cutting forces during machining and that while maintaining the feed per tooth with a reduced number of inserts, the roughness is not significantly affected. An unexpected result was that inserts can differ in terms of the surface quality achieved. This research also shows that individual inserts can vary substantially in the force load they generate, a phenomenon that can be attributed to their dimensional differences. This study provides valuable insights for industrial applications that require precision machining concerning cutting forces and surface quality. It can potentially improve the efficiency and quality of machining in industrial applications.

## 1. Introduction

Manufacturing processes, especially machining, can affect the integrity of a workpiece surface by producing high temperatures during cutting, resulting in plastic deformation in the workpiece (residual stresses), changes in surface geometry (roughness, cracks, deformations), and chemical reactions between the tool and the workpiece [1,2].

Constant changes in cutting forces during the machining process directly affect the process’s stability. They can even damage tools, fixtures, and machine tools [3,4]. Studying the dynamic characteristics of these forces is essential for the control of the milling process from the point of view of performance, quality, and economy [3,5]. Measuring cutting forces during machining processes is thus a fundamental step in determining and controlling the conditions under which the machine, tool, and workpiece work. By measuring cutting forces, it is possible to estimate operating temperatures, tool wear, and energy consumption, i.e., properties important for process improvement [5,6]. Understanding the relationships in the cutting process helps optimize individual process parameters successfully and prevent critical situations in the field of work safety. For specific workpieces (parts made of fragile materials or with thin walls prone to cracking), the limiting factor is mainly the cutting forces [7,8]. The dynamic stress of the machine–tool–workpiece–fixture system caused by cutting forces also has a fundamental effect on the accuracy of the workpiece and surface integrity. Information on the extent of the cutting force and its behavior during the cutting process enables final economic optimization analyses of production processes [5,7,9].

The best machining conditions depend on the cutting tool, workpiece, machine tool, cutting fluids, and cutting parameters, so it is necessary to make experimental measurements. This is because of the problem of choosing from many commercially available tools. The manufacturer’s recommendations should only be used as a guide, as better conditions may be found for other tools and cutting parameters. Testing for each application is very important because of the economic benefits that accrue for manufacturing industries that perform machining operations [4,10,11,12].

Many authors have devoted themselves to investigating cutting forces during the milling process. Chuangwen and his team [13] conducted an extensive study investigating the effect of cutting parameters on the wear of cutting force and vibration during corner milling with a double-edged cutter. Their finding was a decrease in cutting forces with an increase in feed per tooth and cutting speed. On the contrary, increasing the depth of the cut is the cause of growth. According to their conclusions, the depth of a cut has the most significant influence on the intensity of the cutting forces, followed by the cutting speed and the feed per tooth. Yesilyurt [14] designed a force model and subsequently performed an experimental verification of the cutting forces applied in producing gears. Tukora et al. [10] focused explicitly on milling complex shapes. Their prediction system relied on a graphics-processing unit (GPGPU), which, unlike the usual method of acquiring geometric information about the contact area of a workpiece and a tool, obtains information directly from the multi-texel representation of a workpiece, which allowed them to determine the forces at any point of contact.

Flat surfaces are found on almost all components; most require machining to perform their desired function. Such surfaces’ quality also determines a component’s life span. Therefore, research into flat-surface machining is still an urgent task. Different methods can be used for machining flat surfaces: planing, chopping, face milling, broaching, grinding, scraping, or lapping. However, face milling and grinding are the most widely used due to their simplicity. Moreover, most machine parts do not require high-quality grinding surfaces. Therefore, face milling is the most popular because it allows high productivity and is highly accurate [15,16,17]. Heads with replaceable inserts are very widespread. Commercially available heads contain 4, 6, 8, 10, 64, or more inserts [18].

The study of face milling is closely connected with the inserts used. Meng and his team [16] studied face milling. In their work, they investigated energy consumption during face milling using tools with different numbers of inserts. Tan [19] et al. conducted a study of adhesive wear of inserts; one of their findings was that the shape of the grooves plays a vital role in this phenomenon. Mrkvica and Janoš [20] similarly investigated the wear of circular inserts when machining Inconel 718.

None of the studied articles were devoted to determining cutting forces during face milling with variable numbers of inserts used in the milling head. An article by Dilipak and Gezgin [21] addressed the effect on roughness and came to the conclusion that the number of inserts is the most significant parameter. However, they did not adjust the cutting conditions so that the inserts would meet the recommended conditions given by the manufacturer. For this reason, this article was created to understand how cutting forces differ depending on the number of inserts. It will help reveal how individual inserts can affect a workpiece’s force loading and their effect on surface roughness.

## 2. Materials and Methods

The material for the experiment, supplied by Mausburger, was 1.1730 steel. It is an unalloyed tool steel with excellent machinability and good core toughness. It is used for unhardened mold parts (plates and frames) and die sets. Its composition is shown in Table 1.

### 2.1. Cutting Tool

A milling head from the company Seco labeled R217.69-1225.RE-10-4A was used to perform milling. Its four insert slots enabled 1-, 2-, and 4-insert machining with one tool while preserving symmetry. An asymmetric distribution of inserts would cause unwanted vibrations and thus invalidate any measurements. The inserts used were also from Seco, Brno, Czech Republic (XOMX10T308TR-ME07 MS2050).

**Figure 1:**
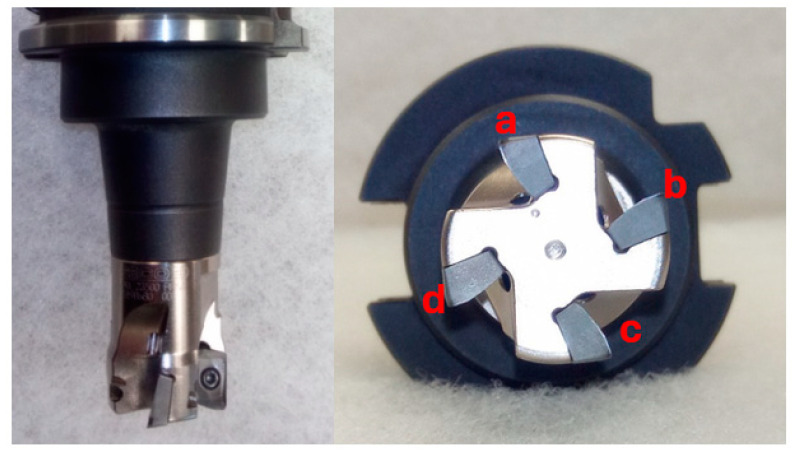
Cutting tool.

To ensure the best possible machining conditions, the inserts used were newly purchased so that their possible wear did not invalidate the measurements. As shown in Figure 1, a short holder was used for machining to minimize possible vibrations. Individual inserts were assigned a designation (a–d), which will be used further in this article for better clarity.

**Figure 2:**
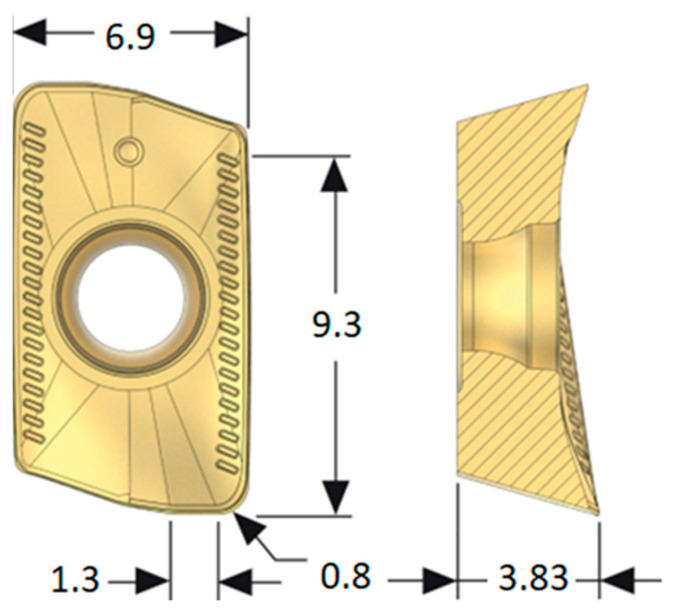
Insert geometry.

The geometry of inserts used is shown in Figure 2.

### 2.2. Machining Tool

**Figure 3:**
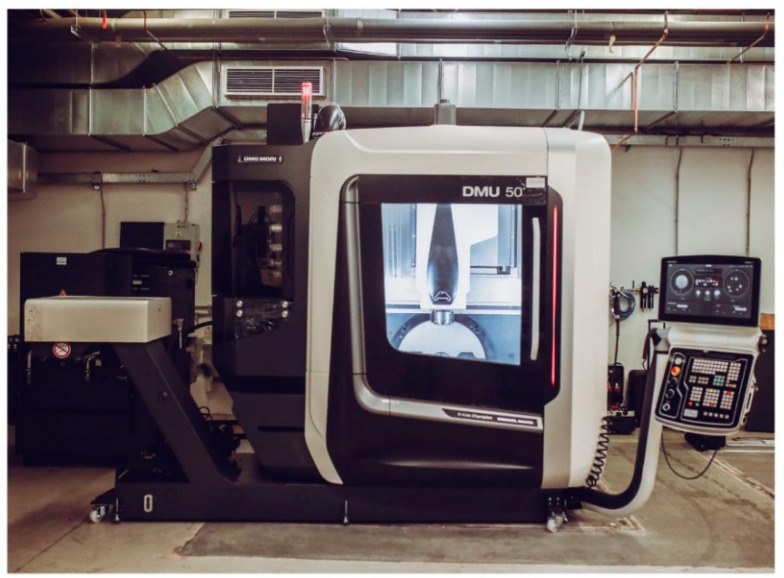
DMU 50.

DMU 50 (Figure 3) from DMG MORI, a five-axis milling center, was used to perform milling. The spindle allows displacements in the *X*, *Y*, and *Z* axes. The clamping table allows rotation in the *B* and *C* axes. The maximum speed of the spindle is 15,000 rpm, and the maximum feed rate is 30 m/min.

### 2.3. Dynamometer

**Figure 4:**
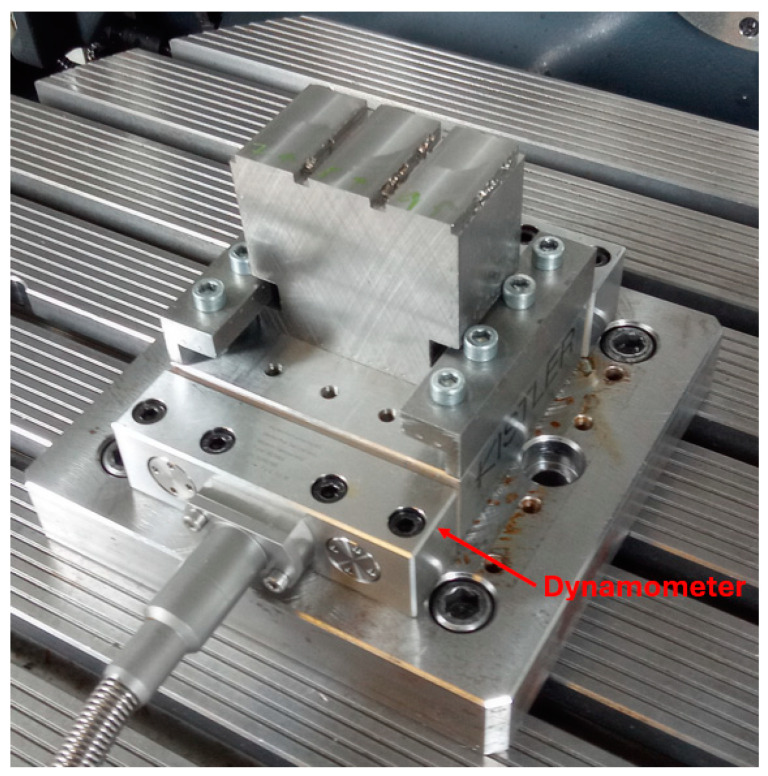
Kistler 9129AA.

A Kistler dynamometer (9129AA) was used to measure cutting forces (Figure 4). The clamping surface dimensions are 90 × 105 mm.

### 2.4. Cutting Geometry

**Figure 5:**
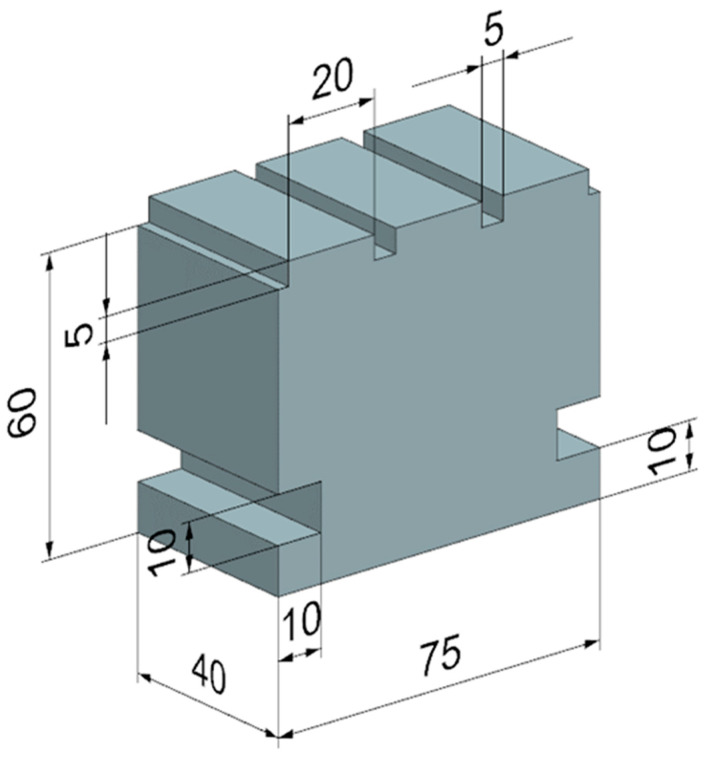
Dimensions of the test body.

Three surfaces with a width of 20 mm and a length of 40 mm were made on the test body. Grooves were created between the individual surfaces to prevent the walls from influencing the machining process. Grooves were also made on the test body to allow it to be clamped onto the dynamometer. The test body is shown in Figure 5.

### 2.5. Cutting Conditions

Nine unique measurements were made. Each of them was repeated 3 times to verify the results. The average values from these measurements are always presented. The conditions and the number of inserts clamped onto the milling head were changed for each measurement to compare individual results. Table 2 shows individual cutting conditions. They were established according to the manufacturer’s recommendations. In order to achieve greater variety, the feed-per-tooth values were changed, as shown below. For the experiment, it was desirable to maintain the same displacement per tooth even when the number of teeth was changed. Below, it is possible to see that the feed changed when the number of inserts changed.

## 3. Results

**Figure 6:**
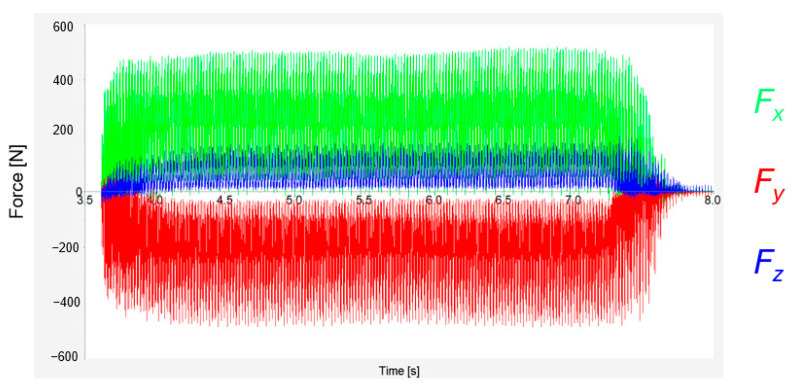
Cutting forces representation.

Figure 6 displays results obtained using Kistler’s Dynamowere software. Blue, green, and red show the loadings on the *X*-axis, *Y*-axis, and *Z*-axis, respectively. It is possible to notice that the results on the *Y*-axis are negative values. The results correspond to the orientation of the dynamometer. For clarity, all results are presented as positive values, as these forces correspond to the tool’s action against the workpiece.

**Figure 7:**
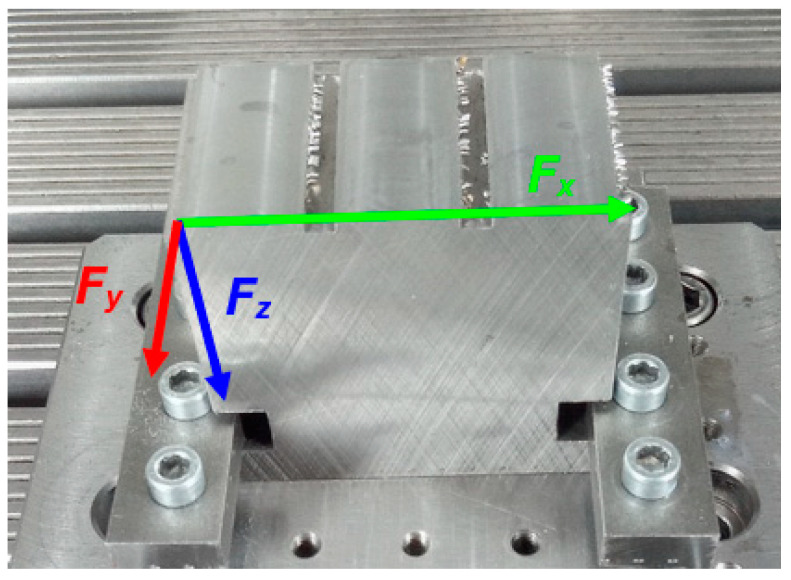
Orientation system of the dynamometer.

The orientation system of the dynamometer is shown in Figure 7. The arrows show the positive directions of the individual axes. For simplicity, the forces in the individual axes are marked with an index indicating the affiliation to the *X*, *Y*, and *Z* axes.

**Figure 8:**
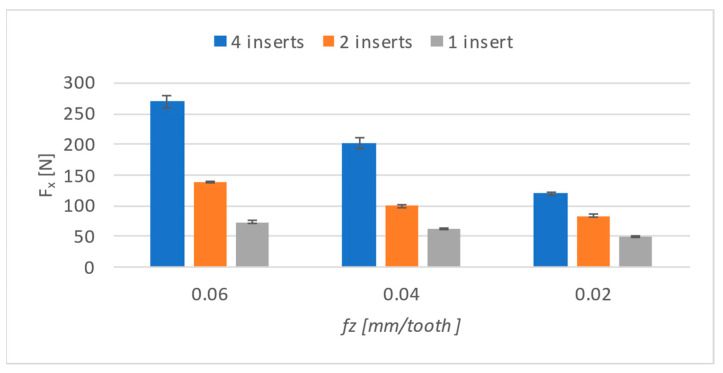
Cutting forces on *X*-axis.

The cutting forces were evaluated by averaging the entire engagement of the tool with respect to its steady part. The values from the individual axis were then entered into graphs. When comparing the results obtained for the cutting force in the *X* axis (as shown in Figure 8), it can be seen that the cutting force on this axis decreases not only with a decrease in feed per tooth but also with the number of inserts.

**Figure 9:**
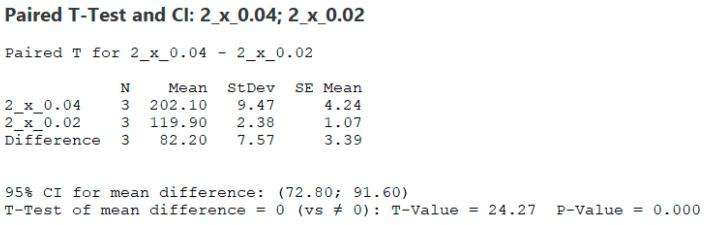
*t*-test.

Furthermore, it is possible to see that the decrease between individual shifts per tooth occurs in a different ratio. In Table 3, the numerical values of the results are given, and a percentage value compared to the highest feed is added to them. Adjacent values, for example, values obtained when machining with four inserts at feed rates of 0.04 and 0.02, were subjected to a paired t-test, and the resulting *p* values are listed below these results. Figure 9 shows how the t-test was performed.

Cutting force, *F_x_*, showed the most dramatic decrease when using all four inserts when the value at the lowest feed-per-tooth rate dropped below half of the value obtained at the highest feed-per-tooth rate. Furthermore, the final values were 60 and 65 percent for two inserts and one insert, respectively.

**Figure 10:**
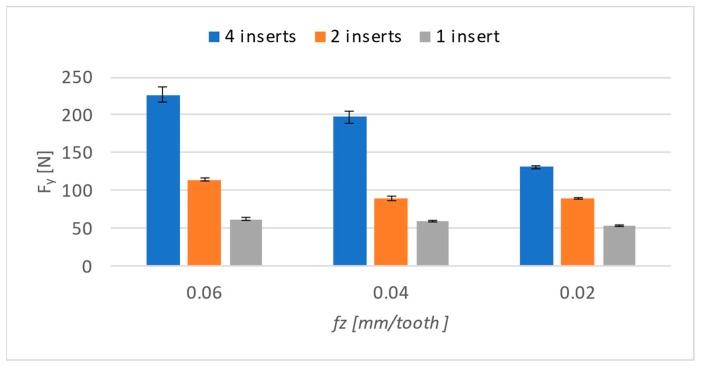
Cutting forces on *Y*-axis.

The results can be seen in Figure 10 for forces on the *Y*-axis, and a different trend can be seen compared to that on the *X*-axis. A significant downward trend is present only for four inserts, and there is only a slight decrease for the rest of the settings.

When comparing the results in the table numerically (Table 4), several changes compared to the *X*-axis can be seen. Not only was there not quite a significant decrease, but the differences were not significant when compared to the *X*-axis. The smaller decrease and the more stable results in this case can be explained by the maintenance of a constant cutting speed, because the machining of the surface took place at a constant feed rate.

**Figure 11:**
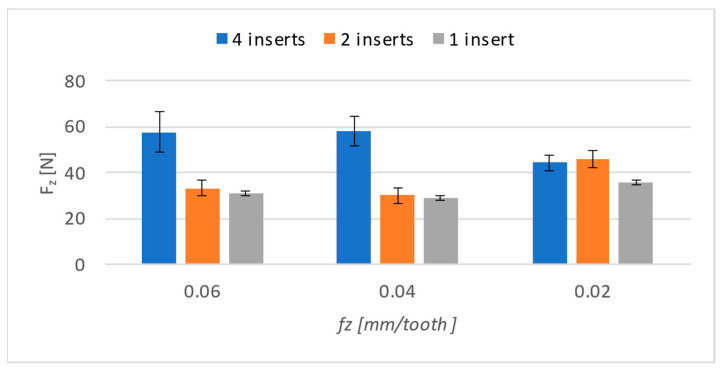
Cutting forces on *Z*-axis.

The results are the most surprising for the *Z* axis (Figure 11). From the graphic display, it can be seen that with 0.06 and 0.04 mm/t, the differences are not significant, and therefore the differences in the feed rate must be greater in order for them to be able to manifest themselves on the *Z* axis.

In comparison to both the graphical and numerical results (Table 5), there is a noticeable deviation from the trends that were present in the previous results. A feed-per-tooth rate of 0.02 (mm/t) yielded results that significantly differ from the results obtained under other conditions. When using two inserts and one insert, there was even an increase in the lowest feed value per tooth.

The significant differences on the *Z*-axis show a possible inconsistency in the parameters set during the measurement. All possible parameters (depth of cut, feed, etc.) were checked, and no discrepancy was found. The last parameter that could have caused this is the inserts used. However, this possibility seems the least likely. The inserts used for measurement were newly purchased, and they were first used for these measurements.

**Figure 12:**
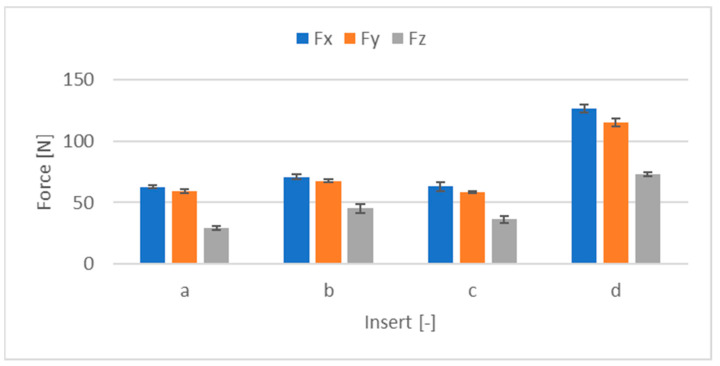
Compression of individual inserts.

In order to determine the possible influence of the inserts, experimental machining of the surfaces was carried out by using individual inserts one at a time. The measurement was performed at a feed-per-tooth rate of 0.04 mm/t, eliminating another possible influence. The inserts were clamped onto the same slot. The results were again averaged and entered into a graph (Figure 12).

From the graph, it can be seen at first glance that there is a significant difference between the individual inserts. The d-marked insert shows much higher cutting forces than the others. Thus, it can be assumed that this insert differed in shape from the others.

**Figure 13:**
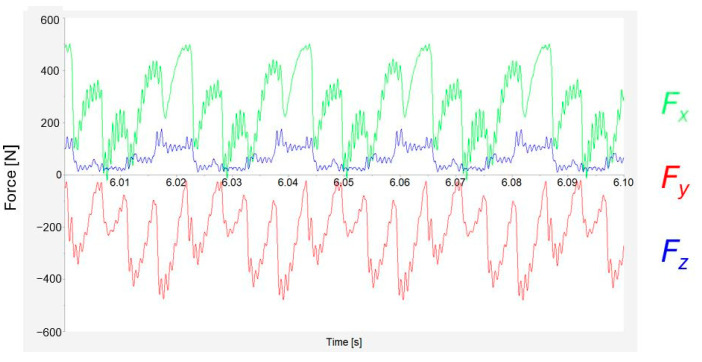
Detail of cutting force record for four inserts.

In the figure examining the recorded cutting force in more detail (Figure 13), only a tenth of a second of the machining process using four inserts and the highest feed-per-tooth rate is shown. The individual engagements of the tool and the differences between them are visible at first glance. For *F_x_* (the green line), individual engagements with the inserts are noticeable. Each peak represents one insert. Every fourth peak reaches higher values than the others. Also, this engagement has the smoothest flow of all. This smoother cutting process could be due to the quality of the surface of the insert itself when such a surface ensures smoother cutting and material removal.

**Figure 14:**
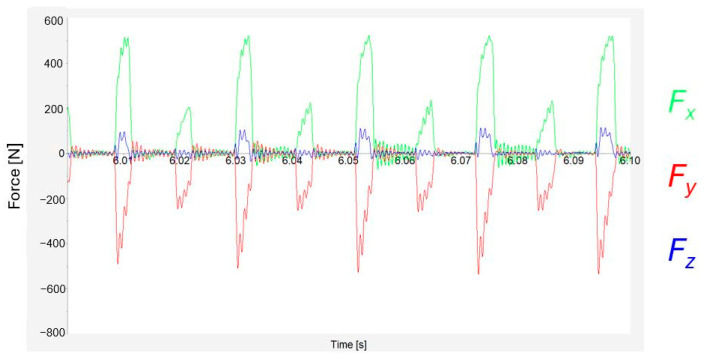
Details on cutting force record for two inserts.

This difference in the course of forces is even more noticeable when using two inserts, marked d and b (Figure 14). Due to the lower number of inserts, the process was discontinued, and pauses are thus visible between engagements. The measured forces that can be seen between engagements are caused by vibrations. At first glance, it is evident that one insert (d) generated up to double the load. Such a significant difference was not expected. This insert likely differed in shape from the others.

**Figure 15:**
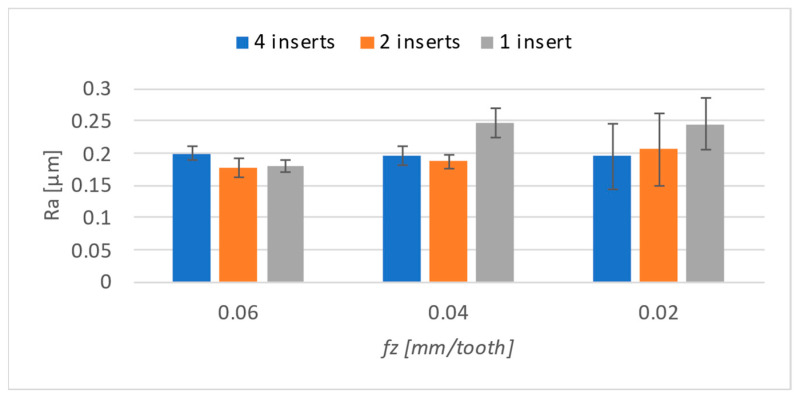
*Ra* results.

In addition to the evaluation of the cutting forces, the surface roughness obtained was evaluated. The goal was to determine if and how the number of inserts in the tool affects the roughness of the surface. Suppose the cutting speed is adjusted so that the feed per tooth remains constant even with a reduced number of inserts used in the tool. Measurement was carried out using a 3D profilometer, and the evaluation area was 3 × 3 mm. The center of the machined surface was always evaluated, as the most stable machining process was expected to take place in this area. The evaluated parameters were *Ra* and *Rz*. The results obtained were entered into a graph (Figure 15) according to the number of inserts used.

No significant differences in roughness parameters were expected because the cutting conditions were set such that a constant feed per tooth would be achieved, leading to similar surface roughness. Although the differences are not significant, the highest roughness was achieved when machining with one insert using the two lowest feed levels. The feed-per-tooth effect was essentially negligible in this case. Although it has a downward trend towards lower values, the difference between them is 0.04 μm, which can be considered insignificant. Also, the standard deviation, shown in the graph by the error bars, increases with the decrease in the number of inserts used.

**Figure 16:**
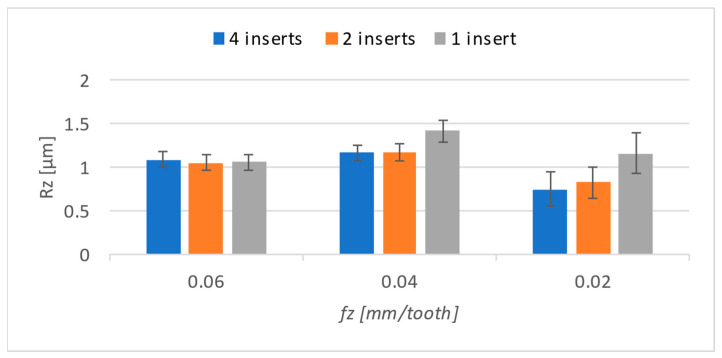
*Rz* results.

The *Rz* results were evaluated identically. The corresponding graph can be seen in Figure 16. Compared to the *Ra* parameter, there was a significant decrease in the feed of 0.02 mm/t for all numbers of inserts. A general recommendation for achieving better surface quality is to reduce the feed rate. Therefore, it is interesting that a significant difference occurred only for the *Rz* parameter. This is also where the most significant deviation occurred at the lowest feed rate. By reducing the feed rate, the friction between the inserts and the already-machined surface lasts longer than it does with higher feeds. This probably creates peaks and valleys on the surface, causing greater dispersion.

**Figure 17:**
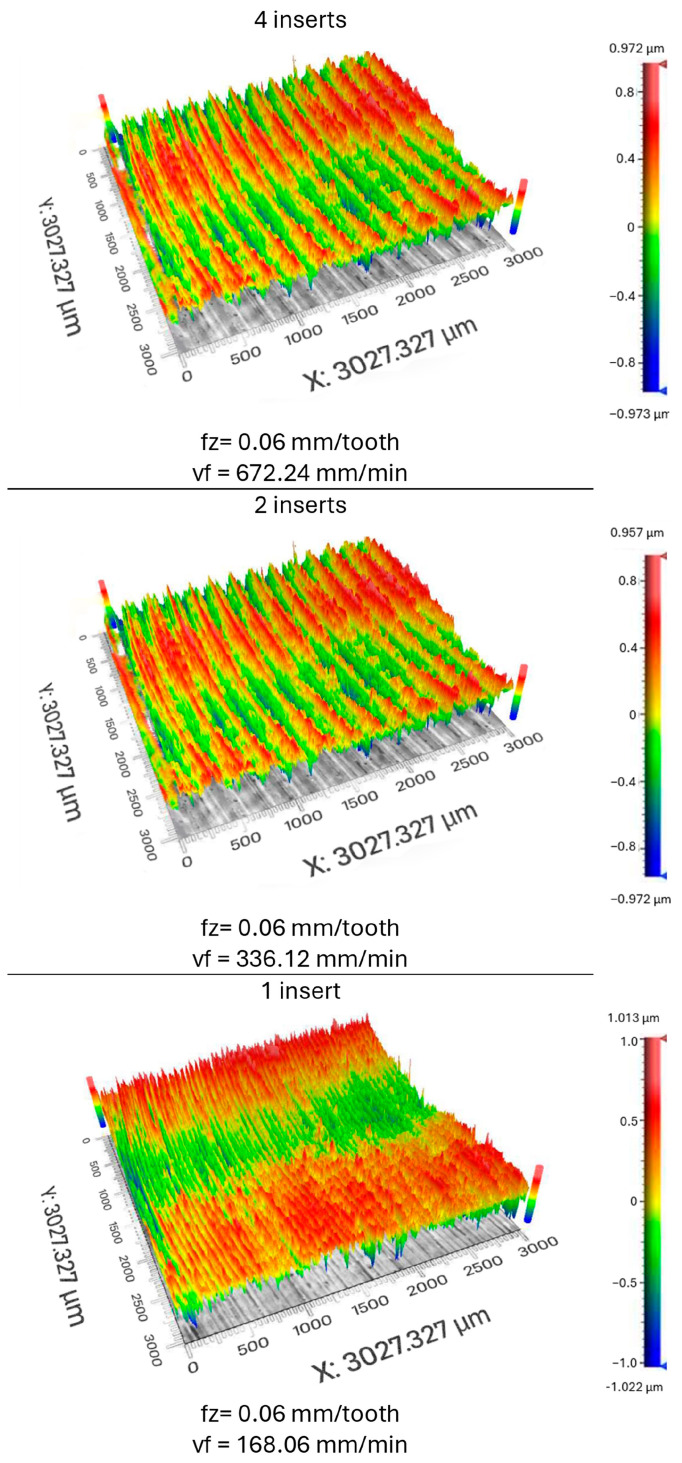
Scans of surfaces obtained by machining 4 inserts, 2 inserts, and 1 insert at *fz* = 0.06 mm/t.

When comparing scans of the individual surfaces (Figure 17), individual traces left by the inserts used during machining can be seen. The scans of the surfaces presented below correspond to those obtained when machining with four inserts, two inserts, and one insert at 0.06 mm/t. It can be seen from the image that the density of valleys and peaks on the surface increases as the number of inserts decreases. This is due to the decreasing amount of feed that was required to keep the feed-per-tooth rate constant. The values of the feeds are given below the images of the scans. It is possible to notice that the values always drop by half because the number of inserts also decreases.

**Figure 18:**
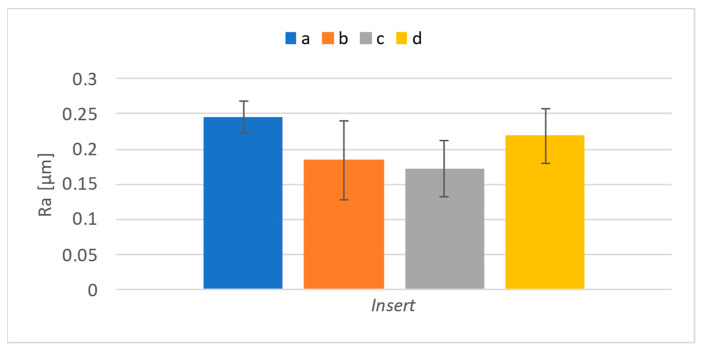
*Ra* results for individual inserts.

**Figure 19:**
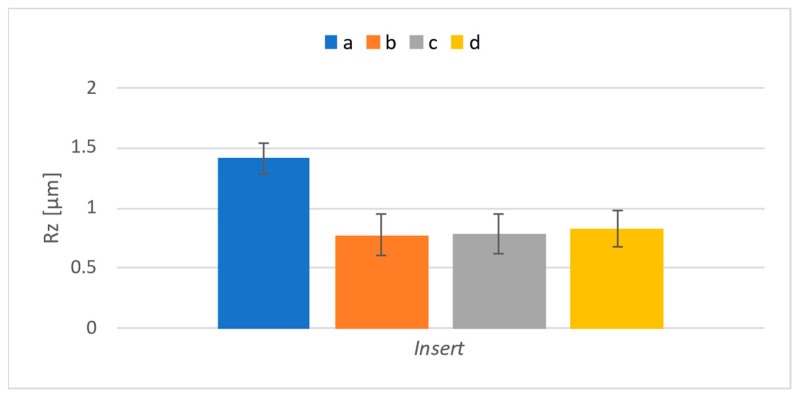
*Rz* results for individual inserts.

Surface roughness was also evaluated for surfaces that were machined with the individual inserts used for the experiment. It was assumed that the difference would also be noticeable in this case. Figure 18 shows the results regarding the parameter *Ra*, and Figure 19 shows those concerning the parameter *Rz*.

The presented results show differences in the examined roughness parameters between the inserts. In the case of cutting forces, the insert marked “d” showed the highest values; in the case of roughness, it is the insert marked “a” that led to the highest roughness. Since the highest roughness was achieved by insert “a”, which had the lowest cutting forces, it can be assumed that insert “a” experienced shape deviation at the corner radius of the insert, due to which the material was not properly cut. On the contrary, insert “d” probably differed in its effective length; thus, a more extensive section of material was removed compared to that for the other inserts, leading to more significant cutting forces. To confirm this, inserts were measured using a presetting device produced by Haimer Micro-vision UNO 20/40. The distance from the base of the device to the top of the inserts was measured to detect any differences in the effective length of the inserts. Furthermore, the distance from the machine’s rotation axis to the insert’s cutting edge was measured to determine any difference in width. Finally, the radius of curvature of the insert was measured. The results are shown in Table 6. Even though the dimension measurements were carried out after the milling, it is unlikely that it could have caused significant wear or even a change in dimensions because the amount of material removed was significantly below the lifetime limit of the individual inserts.

The measurements showed that there were only minor dimension differences between the inserts. The insert marked “d” was indeed the longest, and its radius was the biggest, but the difference in radius is very subtle. However, its length apparently led to changes in cutting forces because a larger amount of material was removed. Insert “a” has the smallest width and radius. It appears that even such differences were sufficient to cause this insert to exhibit greater surface roughness than the others. A visual inspection of it did not reveal any visible difference that could have led to its presentation of the highest roughness value, measured on the surface machined using this insert. The difference in width most likely prevented the stroking surface from functioning properly, which led to the measured differences.

Another possible explanation is that differences in the rake angles of the individual inserts caused the difference in cutting forces. These differences would lead to poor removal of the material from the cutting site, which could then be compacted and increase the cutting resistance, thus increasing the cutting force.

Tool wear seems to be the least likely reason for the differences because, as mentioned above, all the inserts were new. The lifespan of the tools was not exceeded in the experiments performed; therefore, it is unlikely that this was the reason.

## 4. Conclusions

In this article, a study of face milling was carried out to investigate the effect of the number of inserts used in the milling head while changing the feed per tooth. Cutting conditions were adjusted to achieve the same feed per tooth with any configuration of the number of inserts. The feed value was reduced at the same time as the number of inserts. The goal was to determine the cutting force values that could be reached on an individual axis and the degree of surface roughness that could be achieved.

Reducing the number of inserts does not necessarily lead to a significant decrease in cutting force values. Feed is more significant because the cross-section of the removed material decreases during individual engagements. If, in practice, it is necessary to adjust the cutting conditions to reduce the cutting forces, it is for this reason that it is appropriate to choose the path of reducing the feed values. Furthermore, it turned out that the number of inserts had practically no effect on surface roughness. However, this was caused by a feed reduction. If the cutting conditions were maintained with a reduced number of inserts, the quality of the surface would likely deteriorate.

This research also shows that individual inserts significantly differ in terms of the force load they generate and the surface roughness obtainable. Insert “d” produced significantly higher cutting forces than the others. Using the insert marked “a” then worsened surface quality. Therefore, the subject of further research should be the investigation of shape deviations of inserts on the machining process and surface quality. Finding out that individual inserts can produce such differences in the force load and the surface roughness was essential. For example, with strict requirements for surface roughness, an “unsuitable” insert can create defects on the surface, which would then have to be removed, leading to an undesirable increase in production costs. Different force loads could lead to unwanted vibrations, leading to dimensional deviations. In the case of subtle components, they could lead to product deterioration.

## Figures and Tables

**Table 1:** Chemical composition of the material.

	Element Content in %	
Element	Value	Limit Values
C	0.45	0.40–0.50
Mn	0.70	0.60–0.80
Si	0.30	0.15–0.40
P	0.029	≤0.035
S	0.027	≤0.035

**Table 2:** Cutting conditions.

Condition	Value			Unit
depth of cut	2			[mm]
widht of cut	20			[mm]
number of passes	1			[-]
spindle speed	2801			[min^−1^]
number of inserts	4	2	1	[-]
feed per tooth	0.6	0.4	0.2	[mm/t]
cutting speed	220			[m/min^−1^]

**Table 3:** Cutting-force ratios on the *X*-axis.

Feed per Tooth	0.06 [mm/Tooth]	0.04 [mm/Tooth]		0.02 [mm/Tooth]
4 inserts	273.6 [N] (100%)	205.1 [N] (74.9%)		119.9 [N] (43.8%)
*p*-value	0		0	
2 inserts	136.6 [N] (100%)	98.4 [N] (72.1%)		82.3 [N] (60.3%)
*p*-value	0		0.001	
1 insert	75.4 [N] (100%)	60.7 [N] (80.5%)		49.6 [N] (65.7%)
*p*-value	0		0	

**Table 4:** Cutting-force ratios on the *Y*-axis.

Feed per Tooth	0.06 [mm/Tooth]	0.04 [mm/Tooth]			0.02 [mm/Tooth]
4 inserts	229.9 [N] (100%)	200.5 [N] (87.2%)			131.5 [N] (57.2%)
*p*-value	0			0	
2 inserts	112.3 [N] (100%)	88.7 [N] (79%)			88.4 [N] (78.7%)
*p*-value	0		0.738		
1 insert	63.9 [N] (100%)	58.2 [N] (91%)			53.2 [N] (83.3%)
*p*-value	0.011		0.02		

**Table 5:** Cutting-force ratios on the *Z*-axis.

Feed per Tooth	0.06 [mm/Tooth]	0.04 [mm/Tooth]		0.02 [mm/Tooth]
4 inserts	61.5 [N] (100%)	60.9 [N] (99.1%)		42.5 [N] (69.1%)
*p*-value	0.804		0.001	
2 inserts	33.5 [N] (100%)	30.3 [N] (90.3%)		47.3 [N] (141.2%)
*p*-value	0.001		0	
1 insert	29.5 [N] (100%)	24.2 [N] (82.1%)		37 [N] (125.2%)
*p*-value	0.234		0	

**Table 6:** Inserts dimensions.

a	b
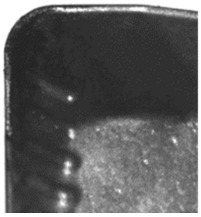	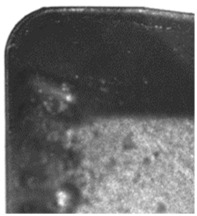
R = 0.75 mm	R = 0.77 mm
X = 12.436 mm	X = 12.471 mm
Z = 89.940 mm	Z = 89.947 mm
c	d
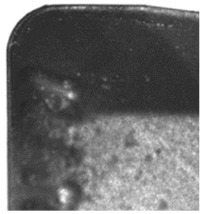	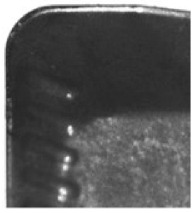
R = 0.77 mm	R = 0.78 mm
X = 12.462 mm	X = 12.477 mm
Z = 89.941 mm	Z = 89.962 mm

## Data Availability

The data presented in this study are available on request from the corresponding author due to privacy.
